# Study protocol to support the development of an all-night binaural beat frequency audio program to entrain sleep

**DOI:** 10.3389/fneur.2023.1024726

**Published:** 2023-01-26

**Authors:** Melisa A. Gantt

**Affiliations:** Gantt Clinical Research Institute LLC, Orlando, FL, United States

**Keywords:** brainwaves, brain entrainment, binaural beat, sleep quality, music

## Abstract

**Background:**

Given that the stages of sleep have specific brainwave patterns, it may be feasible to manipulate brainwaves to induce stages of sleep to improve better sleep quality. Binaural beat frequencies (BBFs) are an auditory-neurologic technique that uses auditory tones *via* headphones to manipulate brainwave activity in turn affecting the listener's state of consciousness. However, BBFs are often sold in only one frequency which may not allow the listener to transition through the phases of sleep. This study is Phase 2 of a four-phase feasibility study to assess if systematically sequencing a variety of BBFs can improve sleep efficiency.

**Methods:**

This protocol uses a two cohort unblinded and double-blinded, randomized, pre- and post-intervention methods and crossover matched group design. In Cohort 1, a sample of 106 participants with poor sleep quality will be randomized into two groups. All participants will start with 1 week of no intervention. Group 1 will use theta/delta BBF for 2 weeks followed by 1 week of no intervention followed by music for 2 weeks. Group 2 will do the reverse. In Cohort 2, 62 participants will be blinded and randomized into two groups. Group 3 will use music for 2 weeks followed by a 1-week break followed by music embedded with theta/delta BBF for 2 weeks. Group 4 will do the reverse. Using Cohort 1 music only as a control, data will be collected using sleep actigraphy, sleep quality questionnaires, and sleep diaries with a crossover and match group analysis between cohorts to compare the effect of no intervention vs. music vs. BBF only vs. music with BBF on sleep quality.

**Discussion:**

Phase 1 concluded that theta BBF was able to decrease stress to help induce sleep. Phase 2 will assess if theta and delta BBFs, with breaks to allow for REM, will be able to sustain sleep to improve sleep efficiency. The data from Phase 1 and 2 will provide information to help construct an all-night audio program with the appropriate BBF and timing to trigger the correct sleep stage for better sleep efficiency. If this concept is feasible, it could be beneficial for many sleep disorders.

## Introduction

### Sleep quality

Sleep is essential as it has a multifaceted connection to health and chronic disease. Due to the rising societal impact of sleep deficiencies and circadian dysfunctions, more research is needed on new strategies to improve sleep quality to support physiological function, behavioral health, and wellbeing throughout the lifespan (1). When adequate sleep is not attained it can negatively affect vigilance, reaction time, learning ability, alertness, mood, hand-eye coordination, and memory (2). In the workplace, it reduces efficiency and productivity, underestimates performance capabilities, and increased errors which can cause accidents (3). According to the 2021 National Sleep Foundation statistics, between 10 and 30% of adults struggle with sleep with 35% reporting sleeping <7 h per night on average with half of all Americans stating that they feel sleepy during the day ~3–7 times a week (4). Given these statistics, over half of adults believe that if they had better sleep, they would be more effective which would positively affect their quality of life (4). Over the years, studies have assessed a variety of evidence-based strategies to mitigate poor sleep quality such as good sleep hygiene, strategic napping, appropriately timed rest breaks, optimal lighting, and pharmacologic (5). However, with all of these strategies poor quality sleep still remains an issue.

### The use of auditory stimuli to improve sleep

The use of auditory stimuli, such as white noise, pink noise, and music, has shown efficacy in improving sleep quality in a variety of populations and settings. In a systematic review published in 2022, the results showed that of 34 studies, 19 studies [6 using white noise (33%), 9 using pink noise (81.9%), and 4 using multi-audio (66.7%)] reported positive effects on improving sleep quality with multi-audio having the lowest risk of bias (mean/standard deviation: 1.67/0.82) compared to white noise (2.38/0.69) and pink noise (2.36/0.81) (6). Another interesting auditory stimulus that has evolved over the last few years is the use of 432 Hz music which is widely used in the New Age genre. This process is done by slowing down (by 32 hundredths of a tone) the execution of a song originally tuned at 440 Hz, using music editing software (7). It is theorized that music played at 440 Hz, which is the current frequency used for tuning musical instruments, has a different psychological and/or physiologic effect than that same music played at 432 Hz (8). In a study published in 2020, 12 spinal injury patients were subjected to their favorite music tuned to 440 Hz or 432 Hz for 30 min each day for two periods (8). Those who listened to music at 432 Hz showed a significant improvement in sleep scores (+3.6, *p* = 0.02) when compared to those listening to music at 440 Hz (−1.50, *p* = 0.34) (8). Another novel auditory stimulus that has been shown to improve sleep quality is the concept known as binaural beat frequencies (BBFs).

### Binaural beats frequencies

**Figure 1:**
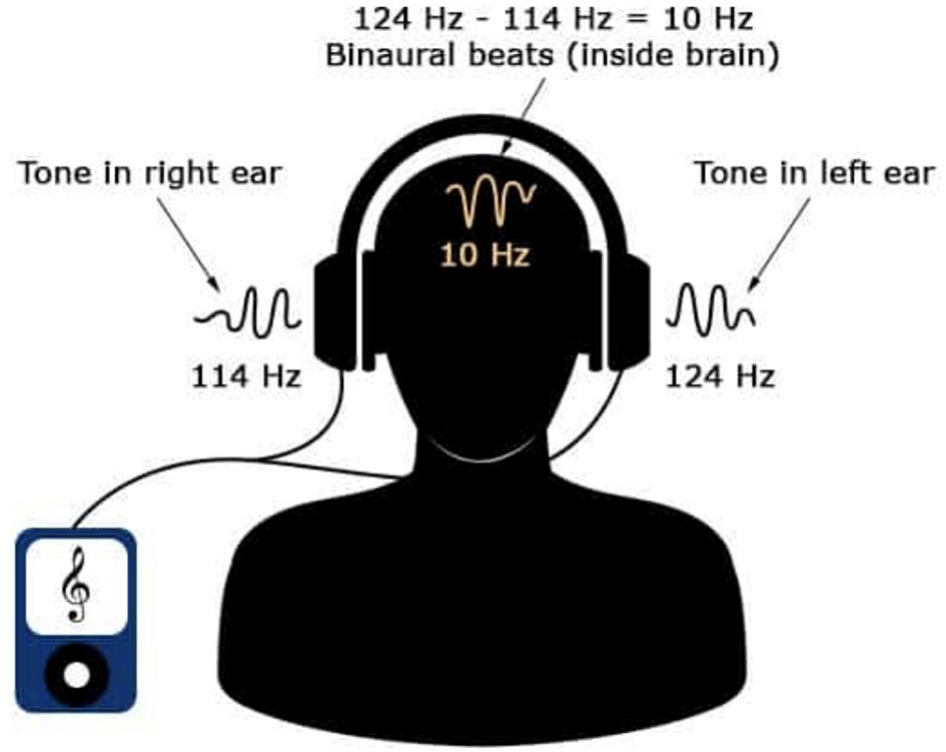
Concept of Binaural beats. Image courtesy of Patrick Alban, DC and Deane Alban. https://bebrainfit.com/binaural-beats-meditation.

The concept of BBF was first discovered by German researcher Heinrich Wilhelm Dove in 1839 (9). This phenomenon occurs when a frequency is played to one ear and a slightly different frequency is played to the other giving the perception of the presence of a third frequency (the difference between the two frequencies) (9) (Figure 1). This third frequency does not reflect a physical property of sound but is generated at the convergence of neural activity from the two ears in the central binaural auditory pathways of the brain (10). This frequency then causes the brainwaves to fall in sync with the frequency in turn altering the listener's state of consciousness (10). “The most widely accepted physiological explanation for this suggests that discharges of neurons that preserve phase information of the sound in each ear according to the volley principle converge on binaurally-activated neurons in the ascending auditory pathway that, in turn, generate brainwaves to fall in sync with the frequency” [(10), p. 1,514–1,515]. Since brainwave frequencies fall into five major categories (e.g., gamma, beta, alpha, theta, and delta) with each producing five distinct levels of states of consciousness, one only has to adjust the sound frequencies to each ear to produce the state of consciousness he/she desires.

Binaural beat frequencies can be constructed using easily accessible audio equipment and software as evidence by the many audio files found on Spotify, YouTube, Apple Music and the Apple app store. However, BBFs are often sold in only one frequency which may not allow the listener to transition through the phases of sleep. Given that commonly used public health web sites are writing about the benefits of BBFs, it is imperative to conduct more research not only for its efficacy but for public safety. According to the National Library of Medicine National Center for Biotechnology Information's database, since 2014 there has been a rise in the number of research studies assessing the efficacy of BBFs due to interest in its potential. Studies have been conducted for its efficacy on *attention* (11), *anxiety* (12–14), *cognition* (15), *mood* (16), *depressive disorders* (17), *stress* (18, 19), with *music therapy* (20), *memory* (21–23), *pain* (24, 25), and even for *post-deployment stress in the United States military* (26).

### The efficacy of using binaural beats frequencies for sleep quality

Although there is a rise in BBFs studies, there are only a handful of published studies assessing the use of BBF for sleep. In a pilot study published in 2013, 15 elite German league soccer players used BBF for 8 weeks to improve sleep quality in hopes to improve performance (27). Once a week participants completed a sleep diary, an adjective list for psychophysical and motivational states, and a self-assessment questionnaire for sleep and awakening quality (27). When compared to a control group there was improved perceived sleep quality and the post-sleep state, however there was no effect on performance which the investigators believed would have required longer exposure to the intervention (27). In a 2017–2018 study, 43 participants with insomnia were exposed to theta BBF and measured using spectral analysis of the quantitative electroencephalography data. When music embedded with theta BBF was played, the relative theta power increased in the occipital lobe (*p* = 0.009) (28). After listening to music with BBF for 2 weeks, the decrease in the beta power from baseline was more prominent than after listening to music without BBF (28). In a study published in 2018, 24 participants were monitored for three consecutive nights (adaptation night, baseline night, and an experimental night) (29). On the experimental night participants were exposed to a 3-Hz BBF on 250-Hz carrier tone (29). While utilizing electroencephalograms, electro-oculograms, and electromyograms, the intervention was initiated when the first epoch of N2 was detected and was stopped when the first epoch of N3 sleep was detected (29). When compared to a control group it showed that the N3 sleep duration was longer and N2 sleep was shorter and did not disturb sleep continuity indicated by arousal index and WASO without sleep fragmentation induction (29). In a 2022 pilot study, 20 students were exposed to 90 min of delta BBF every day for 1 week. When compared to 1 week of baseline, sleep diary and the Profile of Mood State questionnaire showed that the average duration of sleep latency and the number of awakenings were significantly shorter (*p* < 0.001) (30). The mean duration of actual night sleep and waking time in the morning was significantly longer (*p* < 0.001) with the majority of the participants (70%) describing their sleep quality as good to excellent (30).

It is also important to review studies that did not show a significant difference as there may be more information to further investigate or an opportunity to replicate. For example, in a double-blind sham-controlled randomized trial published in 2020, 43 participants with subclinical insomnia were divided into two groups, one using BBF and the other using a sham audio (31). Although the effect was much stronger for the group that used music with the BBF (Cohen d = 1.02 vs. 0.58), the insomnia severity decreased in both groups without significant difference (*p* = 0.656) (31). In reviewing the awake electroencephalographic analysis, the relative beta power was higher in the group that used music with the BBF (0.2 ± 7.02 vs. −3.91 ± 6.97, *p* = 0.041) (31). Taking a closer look at this study, although both groups had a decrease in insomnia, the group that used music with BBF had a higher beta power the next day. It could be possible that using BBF while asleep made the listeners more alert the next day, hence the higher beta power. Another item noted in many of the studies was that only one BBF frequency was used, whereas normal sleep is comprised of transitioning through a variety of brainwave frequencies. Since there is no evidence to support or to reject if using theta BBF (light sleep) alone all night will produce the same or better sleep efficiency than if using it in combination with delta BBF (deep sleep), more studies using multiple BBFs should be conducted.

### Using an ideal hypnogram as a model to construct an all-night BBF audio program

**Figure 2:**
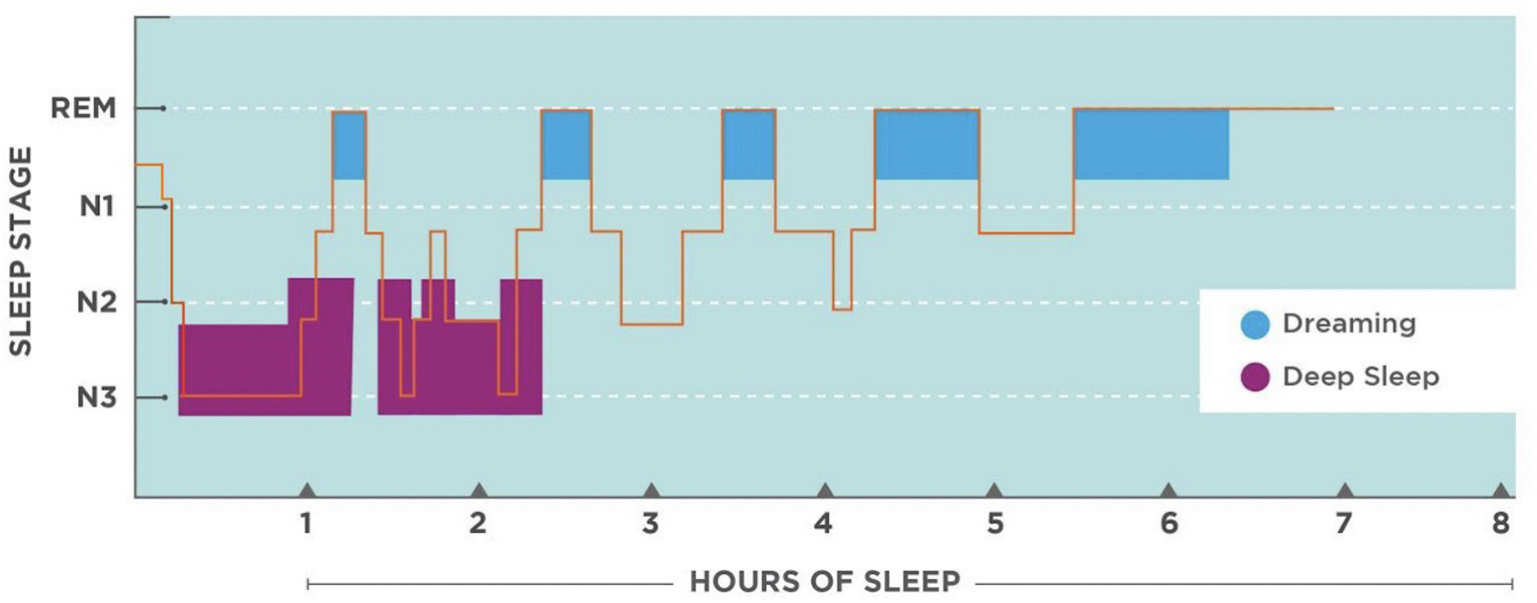
Hypnogram of a normal healthy adult. Image courtesy of Patrick Alban, DC and Deane Alban. https://bebrainfit.com/binaural-beats-meditation.

Studies that have compared hypnograms of healthy adults have formulated some key similarities that can be used as a model of a good night sleep (Figure 2). Although each individual has a variety of factors that make their sleep experience different, using a model of a healthy adult can serve as a starting point that can later be adjusted for the individual. Since the various stages of sleep have signature brainwave frequencies and that BBFs have shown to be able to affect brainwaves, it could be possible to systematically arrange BBFs in an order to reflect a normal sleep cycle and embed them onto a 6-, 7-, or 8-h audio track to entrain an entire night of sleep. Given that poor sleep quality can stem from not being able to fall asleep, stay asleep, or awakening at the wrong time; conducting a series of feasibility studies to address each issue separately can provide data to support the creation of customizable all-night BBF audio programs (Table 1).

**Table 1:** Hypnogram summary and possible ways to use binaural beat frequencies.

**Stage of sleep**	**Percentage of total sleep**	**Timing of occurrence**	**Length**	**Brainwave frequencies**	**Hertz**	**Amplitude**	**Possible ways to incorporate BBFs using an 8-h audio track**
Wakefulness	5–10%	Ends at sleep onset; Brief wakefulness after each REM cycle	Varies	Beta	14–30 Hz	5–20 μV	For a 15 min structured waking at the end of an 8-h audio track, gradually phase in theta BBF for ~5 min. Next, diminish theta BBF and gradually phase in alpha BBF. After ~ 5 min, diminish alpha BBF and phase in beta BBF.
REM	20–25%	~90 min after sleep onset and repeats approximately every 90–110 min	Occurs at the end of 90–110 min cycle and gradually increases ~10% with each subsequent cycle to dominate the last third of an 8-h sleep	Varies	Varies	Varies	Given that REM brainwaves look similar to the awake state, at the 90 min mark of an audio track gradually phase out any BBFs for 30 min (or) simply maintain theta BBF. For every subsequent 90–110 min cycle (~5–6) increase the break by 10–20%.
N1	10%	Sleep onset	1–7 min	Beta to alpha to theta	14–30 Hz to 8–13 Hz to 5–7 Hz	5–20 μV to 30–50 μV to <30 μV	At the beginning of the audio track, gradually phase in alpha BBF. At the 3 min mark, decrease alpha BBF and phase in theta BBF to induce sleep.
N2	50%	Approx. first 30 min of each 90–110 min cycle	Occurs after N1 and after N3	Theta to delta	5–7 Hz to <4 Hz	<30 to >75 μV	Continue theta BBF as in N1. After the first 30 min, decrease theta and phase in delta. Repeat the process every 90–110 min cycle (~5–6), gradually decreasing duration of delta BBF by 10% with each cycle
N3	12–20%	~20–30 min after N1, repeats with each cycle	Occurs after N2 with each 90–110 min cycle and dominates the first third of the night. Gradually decreases ~10% with each subsequent cycle.	Delta	<4 Hz	>75 μV	Continue the alternating of theta/delta BBF with the gradual decreasing of delta BBF as in N2. This will balance out the extended halting of BBFs in the REM state.

## Aims

The primary aim of this study is to assess if an audio program that has theta and delta BBFs arranged in an order that mimics a healthy adult hypnogram will improve sleep quality as evidence by an increase in sleep efficiency score when compared to a control group and no intervention. The author hypothesizes that when similar participants are matched and compared, those using BBF will have similar outcomes.

**Figure 3:**
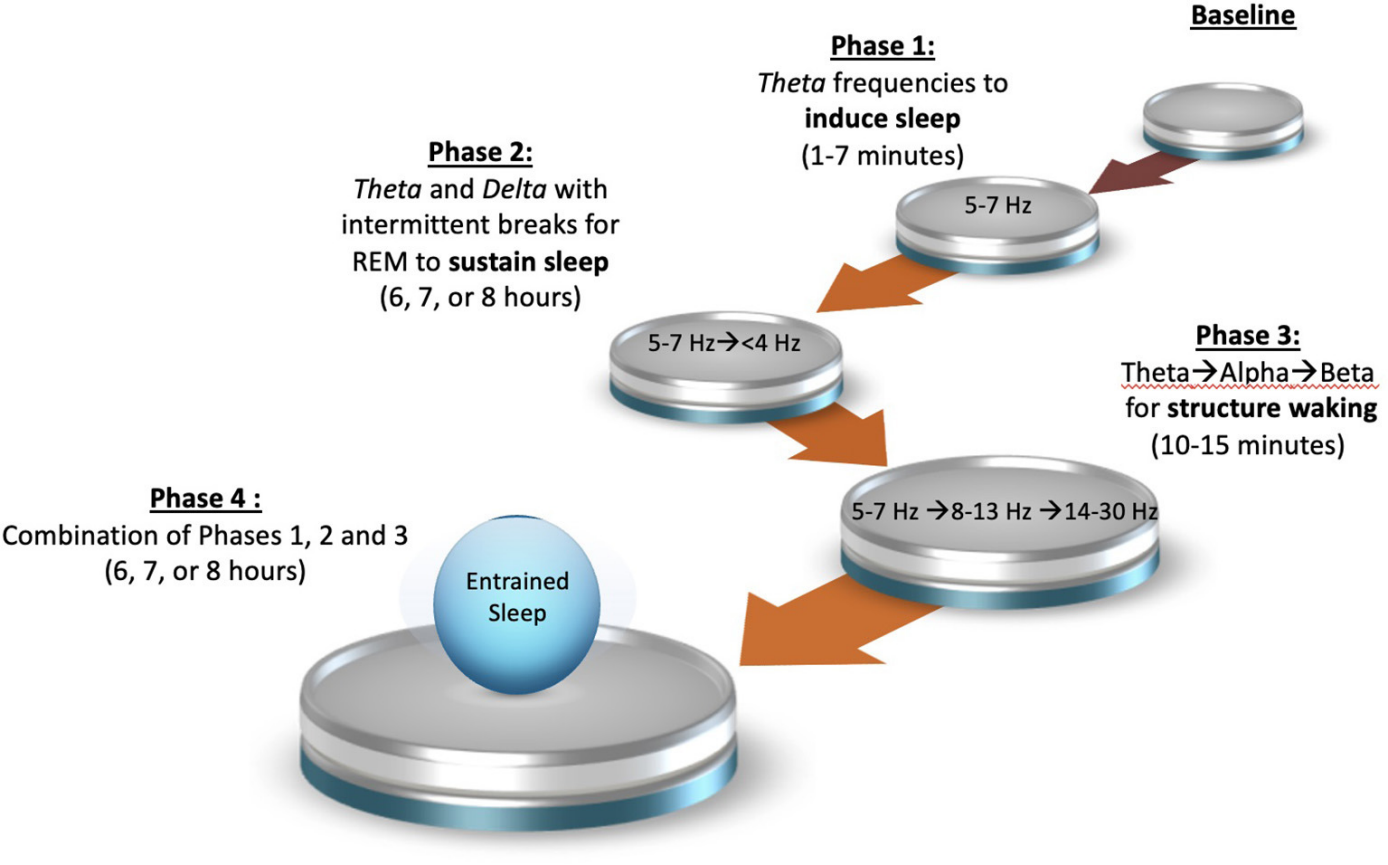
Four phase binaural beat frequency study plan.

Based on the findings of the primary aim, the secondary aim of this study is to map out a plan for a series of future feasibility studies to support the development of a customizable all-night audio program to induce sleep, sustain sleep and provide a structure waking for persons with poor sleep quality (Figure 3).

## Materials and methods

### Study design

The study design for this protocol is based on the findings and unaddressed questions from Phase 1. From 2012 to 2015, the author conducted a Department of Defense funded double blinded study assessing the efficacy of theta BBF in a population of 74 service members with the complaint that post-deployment stress negatively affected their ability to fall asleep (26). Participants were blinded and randomized into two groups, music embedded with theta BBF and the same music without BBF. Each group listened to their respective intervention for a minimum of 30 min at bedtime for at least three consecutive nights per week for a total of 4 weeks. To test the effect of using BBF, participants were subjected to a 20-min pre and post-psychological stressor while wearing a heart rate variability (HRV) monitor and were asked to complete daily diaries to measure perceived stress and sleep. Results showed that 80% of the participants, regardless of group assignment, perceived that they fell asleep quicker than usual. However, when assessing daily stress levels, the group using music with BBF consistently scored lower than the music only group across all time points. Objective measures also showed that the group using music with BBF had a decrease in low frequency HRV (sympathetic drive) (*p* < 0.01) and an increase in high frequency HRV (parasympathetic control) (*p* < 0.01) whereas the group using music alone had the opposite effect. Phase 1 showed that theta BBF did in fact decreased stress, which is important to falling asleep, but its direct relationship to sleep latency could not be confirmed nor rejected which sets the stage for this study which will now reassess theta BBF's effect on sleep latency and theta/delta BBF's effect on sleep efficiency.

Since studies have also shown that music alone can positively impact sleep, Phase 2 will assess sleep in four ways: (1) with no intervention, (2) using music alone, (3) using theta/delta BBF alone, and (4) using music embedded with theta/delta BBF. To do this, two cohorts will be used in a crossover repeated measure matched group design. Although polysomnography is the gold standard for assessing sleep, given that at least 7 weeks of data will be needed, data collection will be done in the privacy of the participant's home using sleep actigraphy and various tested sleep questionnaires.

#### Cohort 1 (n 106) unblinded

Group 1: Baseline → theta/delta BBF alone → 1 week break → music alone
Group 2: Baseline → music alone → 1 week break → theta/delta BBF alone

Since the participants in Cohort 1 will know when they are receiving the intervention, it could be argued that their outcomes are due to a placebo effect, therefore a second cohort using a different group of participants will be used.

#### Cohort 2 (n 62) blinded

Group 3: Baseline → music alone → 1 week break → music embedded with theta/delta BBF
Group 4: Baseline → Music embedded with theta/delta BBF → 1 week break → music alone

Upon completion of both cohorts, a matched group analysis will be conducted to assess the differences between no intervention vs. music alone vs. theta/delta BBF alone vs. music embedded with theta/delta BBF.

### Participants

The target population for this study are persons with complaint of poor sleep quality as defined by the individual's self-satisfaction with all aspects of their sleep experience. To be included in the study, participants must: (1) score ≥13 on the Insomnia Severity Index (ISI), (2) be age 18 years and older, (3) be able to read and speak English, and (4) be able to commit for 7 weeks. Participants are excluded from the study if they: (1) are taking any type of medication that causes drowsiness, (2) have a history of epilepsy, (3) are taking any anti-seizure medication, (4) are incapable of adhering to the research protocol as instructed, (5) have ear trauma, difficulty hearing, or wear a hearing aide, (6) are currently using BBFs (^*^If participant is currently using music or white noise they will be asked to abstain from use while in the study), and (7) suffer from sleep apnea.

### Intervention

Three audio files will be used as the interventions: (1) music alone, (2) BBF alone, and (3) music with BBF. Using an example of a normal healthy adult sleep hypnogram as a model (Figure 2), a brainwave frequency algorithm will be created to reflect brainwave time points and duration (Table 1). The algorithm will then be used to generate dichotic tones to create theta and delta BBFs using *Adobe Audition software*. For example, to create a 6 Hz (theta BBF), a 230 Hz tone track will be generated for the left ear and a 236 Hz tone track will be generated for the right ear and played at the same time. A mini audio segment containing the two tracks made for each BBF will be placed into its correct order and time duration. The segments will then be stitched together to create a succinct 90-min audio file as REM sleep often begins at the 90-min mark. The audio will start with no BBF and will gradually incorporate theta BBF to delta BBF. Five-six 90-min audio tracks will be stitched together with each intro (no BBF) extended by 10–20% to replicate the increase of REM sleep as the night progresses. To create the music with BBF audio file, the BBF audio file will be layered onto a stereo music file (*Natural Healing Society's “100 Royalty Free Meditation and Ambient Songs”*) that has been stitched together to play for a continuous 8 h. The music audio files use textural layers of sound that include nature soundscapes, acoustic instruments such as the piano, strings and flute emulated through a synthesizer. Nature Healing Society's music was chosen because of their inventory of 432 Hz, 528 Hz, and Solfeggio frequency music. A *Soundcraft Signature 12 MDK mixing desk* will be used to balance decibels, to remove artifacts, fade BBF changes, and to set BBF levels to 20–30% below the volume of the carrier sound to minimize detection. Each intervention will be loaded onto a designated *Hotechs 32GB Memory MP3 player* and set to play in a continuous loop until the listener presses stop. The audio will be delivered through *SleepPhones*, a specialized headphone that wraps around the head which contains adjustable left and right speakers. The speakers are connected to one wire which runs down the back of the head and neck as to not to obstruct sleep. All equipment and music are marketed to the general public and have had no safety issues reported to date.

### Data collection instruments

Since the purpose of the study is to assess improvement in sleep quality over repeated measures using a variety of interventions, objective and subjective sleep measures will be collected using the following instruments:

#### Participant demographic questionnaire

Since there are many personal factors that can affect sleep, the Participant Demographic Sheet will be used to capture the descriptive data of the study population. This 9-item form was created by the investigator to capture the participant's age, race, ethnicity, gender, marital status, occupation, normal work hours, average number of hours worked, and perceived sleep quality issue.

#### Insomnia Severity Index questionnaire (ISI)

Given that poor sleep quality is subjective and can present in a wide range, the investigator will use the Insomnia Severity Index as a screening tool to quantify the severity level of the poor sleep quality. The ISI assesses seven items: severity of sleep onset, sleep maintenance, early morning awakening problems, sleep dissatisfaction, interference of sleep difficulties with daytime functioning, noticeability of sleep problems by others, and distress caused by sleep difficulties using a 5-point Likert scale (32). The items are scored from 0 (no problem) to 4 (very severe problem) to yield a total score from 0 to 28. Total scores are categorized into four severity levels: absence of insomnia (0–7); sub-threshold insomnia (8–14); moderate insomnia (15–21); and severe insomnia (22–28) (32). In a 2013 study examining the psychometric indices of the ISI to identify insomnia in 410 adult patients, the instrument had an excellent internal consistency (Cronbach's alpha 0.92) with each individual item showing adequate discriminative capacity (r 0.65–0.84). The area under the receiver operator characteristic curve was 0.87 and suggested that a cutoff score of 14 was optimal (82.4% sensitivity, 82.1% specificity, and 82.2% agreement) for detecting clinical insomnia (33). For participants to be included in the study, they must be within the upper limits of sub-threshold insomnia with a score at least 13 on the ISI.

#### Pittsburgh Sleep Quality Index questionnaire (PSQI)

Given that actigraphy distinguishes wakefulness from sleep based on movements and that there is a possibility that a person with poor sleep quality may stay awake while lying still, the Pittsburgh Sleep Quality Index questionnaire will be used. The PSQI is the most commonly used measure for subjective self-report sleep quality. In a study that examined test-retest reliability in a sample of 76 individuals with insomnia over a period of 2 days to several weeks, the PSQI showed a correlation of 0.87 for global score (34). This 24-item questionnaire captures “overall” subjective sleep quality and will be used before and after each intervention. This instrument gives an accumulative score based on seven subcomponents: subjective sleep quality, sleep latency, sleep duration, habitual sleep efficiency, sleep disturbances, use of sleeping medication, and daytime dysfunction (35). The total score ranges from 0 (good sleep quality) to 21 (poor sleep quality) with a 0–3 scoring range for each of the seven subcomponents (35). To assess significance, baseline scores will be compared to music alone, BBFs alone, and music with BBF.

#### Philips Actiwatch Spectrum Pro actigraphy monitor

Since this study will not be conducted within a sleep laboratory using polysomnography, the Philips Actiwatch Spectrum Pro monitor will be used for home use. This clinical grade tri-axial accelerometer-based wearable device works by measuring movement of the body. Using special software, that information is then articulated into a graphical and tabulated report that shows the movement that occurred throughout the wake and sleep cycles. The report gives the: (1) time the participant went to bed, (2) time awaken, (3) number of hours in bed, (4) total number of hours asleep, (5) number of minutes it took to fall asleep, (6) sleep efficiency percentage, (7) number of minutes awaken after sleep onset, and (8) total number of times awaken after falling asleep. Measures will be collected during all phases. All phases will be analyzed to assess the trends and any statistical differences across time. The device also measures level and type of light exposure in the event the investigator wants to assess if light exposure played a role in the analysis given that some of the participants may work varying shifts (e.g., sleeping at night vs. sleeping during the day) (36). In a 2020 systematic review assessing the utility of sleep actigraphy, the Actiwatch was one of the most used devices with one third of the 34 studies reviewed reporting high participant compliance and low rate of device malfunction (4–6.7%) (37).

#### National Sleep Foundation sleep diary (version 6)

The National Sleep Foundation Sleep Diary is a short 15-item questionnaire that captures the daily habits that may influence sleep. The questionnaire is divided into two parts, seven questions to be completed upon awakening and eight questions to be completed at bedtime. Given that most of the questions upon awakening will be captured by the actigraphy monitor, these questions will not be utilized for the study. As for the questions to be completed at bedtime, these address: (1) exercise, (2) medication use, (3) napping, (4) sleepiness during the day, (5) mood, (6) alcohol, food and caffeine intake, (7) timing of caffeine intake, and (8) bedtime routine. This instrument is not scored but will be used to assess patterns and if those patterns affected the sleep outcomes (38). According to the literature, it is recommended that persons wearing actigraphy monitors also use a sleep diary as it captures subjective data that an actigraphy monitor cannot detect (39).

#### Post-study questionnaire

The Post-Study Questionnaire is a 10-item multiple choice and open-ended questionnaire designed by the investigator to capture subjective data regarding the participant's experience and perception of BBFs. This data will be used to improve the rigor and participant reliability for Phases 3 and 4. Questions include: (1) one word description of their experience, (2) perception if they were able to distinguish if they were receiving BBFs (applies to Cohort 2 only), (3) adherence to the protocol, (4) factors that affected adherence, (5) ease of use of the equipment, (6) level of comfort while using the equipment, (7) willingness to explore other uses for BBFs, (8) willingness to recommend BBFs to colleagues, friends, and family, (9) willingness to participate in a future BBF study in the confines of a sleep lab, and (10) comments or recommendations to the investigator.

### Power analysis and data analysis plan

For Cohort 1, since the study is comparing three measures, a 2 × 3 mixed ANOVA (between and within subject factor) will be used. The parameters for this analysis were as follows: (1) power of 1—β = 0.85, (2) level of significance of α = 0.05, (3) an autocorrelation parameter (i.e., average correlation) ranging from 0.3 to 0.7 (in increments of 0.2), and (4) a standardized effect size (F) ranging from small (0.1) to medium (0.25) in 0.05 increments. Using GPower software, the sample size estimates range from *n* = 52 (when the correlation of the repeated measures = 0.7 and the standardized effect size is *F* = 0.25) to *n* = 116 (when the correlation of the repeated measures = 0.3 and the standardized effect size is *F* = 0.1). For this study a sample of 106 will be sufficient (84 required, plus 25% for attrition bringing the total to 105 and the addition of 1 to make both arms equal in number).

For Cohort 2, assuming there is no difference in mean sleep efficiency score (i.e., mean difference = 0) between the interventions (BBF only, music with BBF) a sample size of 61 participants (plus 1 to make group equal in number *n* = 62) would be needed to declare with at least 80% power that the intervention effect in BBF only is the same as in music with BBF. Given that Cohort 1 is *n* = 106 and that propensity score matching with replacement will be implemented to make the groups comparable, the final test for intervention effect will be carried out with a power in excess of 80%.

For this two-period 2-arm cross-over design where each subject gets both treatments and repeated outcome measurements over time, a linear mixed model with repeated measures (MMRM) approach will be used. Unless otherwise stated, all statistical tests will be 2-sided with the probability of a Type I error not to exceed 0.05. Descriptive statistics will be used for all quantitative and qualitative variables at all data collection points. Continuous variables will be tabulated using N, mean, standard deviation or standard error (as appropriate), median, minimum, and maximum values. For categorical variables, frequency counts and percentages will be presented.

Actual values and change from baseline in the different Sleep Actigraphy report outcome variables will be summarized by time point and sequence group using descriptive statistics. Analysis will be conducted for the following variables: number of hours in bed, total number of hours asleep, number of minutes it took to fall asleep, percent efficiency of sleep, number of minutes awaken after sleep onset, and total number of times awaken after falling asleep. Sleep efficiency will be used as the main indicator for improvement of sleep quality and will be calculated by dividing the amount of time spent asleep (in minutes) by the total amount of time in bed (in minutes) and multiplying it by 100. Study parameters include sleep latency <30 min, wakefulness after sleep onset <30 min, decreased frequency in number of awakenings, total sleep time >6 h and sleep efficiency >80%.

Qualitative measures of sleep obtained from the PSQI will be analyzed using descriptive statistics. As for the National Sleep Foundation Sleep Diary entries, summary statistics will be provided at the different data collection time points. To further improve the precision of the estimates, items from the National Sleep Foundation Sleep Diary scores and light exposure (sleeping at night vs. sleeping during the day) may be entered into the model as additional covariates. To assess if the participants' personal characteristics' affect the outcome a two-step modeling approach will be followed. First, the effect of each demographic variable will be evaluated separately in a series of separate MMRM models that include each study outcome as the dependent variable and the demographic characteristic and time as predictors. All demographic factors will then subsequently be entered into a MMRM model that also incorporate intervention, period, sequence as fixed effects, and study participant as a random effect.

For the match group analysis between Cohorts 1 and 2, propensity score matching will be implemented to construct a sample of subjects which have similar baseline covariate distributions between the cohorts. A logistic regression model, with intervention cohort as the dependent variable, will be employed to estimate the conditional distribution of the intervention given the covariates. The predictors in this model are baseline characteristics believed to be related to the main study outcome of interest (e.g., sleep efficiency score). To make optimal use of the number of subjects enrolled in Cohort 1, a match and replace method will be used whereby a BBF embedded with music user could be matched to more than one BBF only user. This will help maximize the number of matched BBF participants while enhancing the ability of the study to detect a difference in intervention between the two cohorts if indeed there is one.

### Study procedure

#### Screening, consenting, and randomization process

Once the participants have been screened using the inclusion and exclusion criteria, they will sign an Institutional Review Board approved consent and randomized into two cohorts using block randomization. This process was chosen as it reduces bias and increases the probability that the group in each cohort will contain an equal number of individuals (4). Participants in Cohort 1 will then be randomized into Groups 1 or 2 while participants in Cohort 2 will be randomized into Groups 3 or 4.

#### Initial appointment

Participants will be asked to complete the Participant Demographic Sheet and PSQI questionnaire. At the completion of the appointment, each participant will be given:

1) Sleep actigraphy monitor.
2) Instructions on how and when to complete the National Sleep Foundation sleep diary questionnaire that they will receive daily *via* email. Participants will be instructed to complete diary entries within 24 h of receipt. Data will be uploaded anonymously in real-time to the online survey dashboard.
3) One MP3 player pre-loaded with the designated audio file based on group assignment.
4) One set of SleepPhones stereo headphones to work in conjunction with the MP3 player. If a participant experiences discomfort with the SleepPhones, personal headphone or earbuds may be used at his or her own risk as long as it emits stereo sound and does not have noise canceling technology.

#### Weeks 1 and 2—Baseline

For the first 2 weeks participants will be instructed to wear the actigraphy monitor and complete the daily sleep diaries to obtain baseline data.

#### Weeks 3 and 4—Intervention 1

Participants will be instructed to use their designated MP3 player at bedtime. Once the play button is pressed, the audio file will automatically play a continuous loop throughout the duration of their sleep. Participants will continue to wear the actigraphy monitor and complete the sleep diaries as in Weeks 1 and 2. If participants find that the audio file prohibits them from sleeping, they are to discontinue its use and to contact the investigator within 24 h. The MP3 players will come with an accompanying USB cable to allow for the participants to charge the device if needed. The actigraphy monitors, which can hold a charge up to 30 days, will be charged by the investigator. In the unlikely event that the monitor fails, whatever data is collected will still be used. However, since it is unclear if there is a dose effect for BBF or music for that matter, participants will not be asked to recapture any missing data as it could impact the outcome measures. If a participant forgets to wear the monitor, they will still be required to complete the sleep diary questionnaire and note it in the questionnaire.

#### Appointment 2—Equipment exchange

At the conclusion of Week 4 participants will be scheduled for an appointment to exchange the sleep actigraphy monitor and MP3 player and complete the PSQI for Intervention 1. Before departing, participants will be given:

1) A newly charged sleep actigraphy monitor.
2) One MP3 player pre-loaded with the designated audio file based on group assignment.

#### Week 5—Break

For the next week, participants will be instructed to abstain from using any audio stimulation at bedtime but to continue to wear the sleep actigraphy monitor and to complete the daily sleep diaries.

#### Weeks 6 and 7—Intervention 2

The investigator will contact the participant to instruct them to commence using their second intervention. Just as in Weeks 3 and 4, participants will use their respective audio intervention while wearing the actigraphy monitor and completing the sleep diaries. If the music or sound technology prohibits the participant from sleeping, the participant is to discontinue its use and contact the investigator within 24 h.

#### Final appointment

The participant will be scheduled to return all equipment. The investigator will check the online survey dashboard to ensure that all sleep diaries have been completed. Participants will then complete the PSQI for Intervention 2 and Post-Study Questionnaire (Table 2).

**Table 2:** Data collection procedure.

**Action**	**Initial appointment**	**Weeks 1 and 2**	**Weeks 3 and 4**	**2nd appointment**	**1 week break**	**Weeks 6 and 7**	**Final appointment**
Screening, consent and randomization	X						
Participant demographic questionnaire	X						
National Sleep Foundation sleep diary		X	X		X	X	
Philips Actiwatch Spectrum Pro actigraphy monitor		X	X		X	X	
MP3 player with intervention 1			X				
MP3 player with intervention 2						X	
Pittsburgh Sleep Quality Index	X			X			X
Post-study questionnaires							X

### Outcomes measures

The primary aim of this study is to assess if an audio program that has theta and delta BBFs arranged in an order that mimics a healthy adult hypnogram will improve sleep quality. To subjectively measure improvement in sleep quality, the PSQI total scores from Interventions 1 and 2 will be compared to baseline to note any statistically significant differences. This score will also be correlated with the sleep efficiency percentage scores taken from the sleep actigraphy monitor as a secondary validation. To objectively measure improvement in sleep quality, sleep efficiency percentage calculated from the sleep actigraphy monitor will be used. Even though 80 to 85% is considered good sleep efficiency, improvement in sleep efficiency will be determined by identifying any statistically significant changes when compared to the participant's baseline where there was no intervention. Sub measures of sleep efficiency such as sleep latency, number of minutes awake after sleep onset, and number of times awaken throughout the night will be analyzed individually. Data collected from the National Sleep Foundation Sleep Diary will be assessed to see if there are any relationships between exercise, medication use, napping, sleepiness during the day, mood, alcohol, food and caffeine intake, timing of caffeine intake, and bedtime routine and the various sleep outcomes. The Post-Study Questionnaire will be used for informational purposes to assess the participants' experience, perception of the technology, and the feasibility of conducting Phases 3 and 4.

Based on the findings of the primary aim, the secondary aim of this study is to map out a plan for a series of feasibility studies to support the development of a customizable all-night audio track to induce sleep, sustain sleep and to provide a structure waking. First, if Phase 2 shows that the theta/delta combination with appropriate breaks for REM and timing did not sync appropriately due to each participants' unique sleep cycles, this step will be repeated using theta BBF only as theta will allow participants to fall back to sleep in the event they have a longer sleep latency or if awaken throughout the night. If Phase 2 shows efficacy with using the theta/delta combination, Phase 3 will next incorporate theta, alpha and beta BBFs, respectively, at the end of a 6-, 7-, or 8-h audio track to assess if it can trigger a structured waking process. For this phase the attention will be focus more on the ending of sleep such as awakening time and feeling upon awakening. In this case it may not be necessary to use BBFs for the entire night but to simply add a structured waking protocol near the end of an audio track. If Phase 3 proves successful, Phase 4 will combine lessons learned from Phases 1, 2, and 3 to create a customized all-night audio track starting with alpha and theta BBFs, that will transition to intermitted halting of theta and delta BBFs, and end with theta, alpha and beta BBFs. The ideal participant for this study would be persons with insomnia with the hope of using this technique later for persons with sleep phase disorder, non-24-h circadian rhythm disorder, irregular sleep wake disorder, shift work disorder, and/or jet lag disorder. Given that close assessment of sleep stages will be imperative to determine if the brainwaves are in fact being entrained, this study would be conducted within a sleep lab. According to Jirakittayakorn and Wongsawat (29), it may be possible to use electroencephalograms, electro-oculogram, and electromyograms without full polysomnography as they are sufficient for scoring sleep stages and offer better comfort for participants. Although this will be time consuming, equipment intensive, and would require much from the participant, a substantially smaller sample size may be adequate if data from the previous phases were found to be significant. If sleep actigraphy data from the previous phases proved to be consistent and reliable, Phase 4 could also include a hybrid approach of using actigraphy for 1 week and polysomnography later to truly assess if entrainment is taking place.

## Discussion

As evidence of the many studies that have been conducted and the increase interest in BBFs, there is enough evidence to support the possibility of using BBFs to entrain a full night of sleep. Although polysomnography is the gold standard for assessing sleep stages and sleep quality, conducting small feasibility studies will provide valuable information for a more comprehensive study as each phase builds on its predecessor. Studying the three transition points of sleep (e.g., falling asleep, staying asleep, and awakening) individually instead of assessing an entire night of sleep provides more data for customization especially if using the same participants in subsequent phases. When considering the use of BBF in future sleep research there are certain things to consider. First, since brainwaves in REM look similar to the awake state, this could be difficult to replicated as incorporating beta BBF may abruptly awaken the person. Halting BBFs at the end of a 90–110-min sleep cycles to allow for REM may have a better effect than the continual use of delta BBF. If delta BBF is used throughout the entire sleep cycle, it may prohibit the person from entering REM which could negatively impact sleep quality. An example would be REM rebound when BBFs are not in use. This may cause the person to experience an unusual succession of dreams, which may or may not be good for persons with certain mental health issues. Second, age must be taken into consideration when creating a BBF algorithm as increased age may show a decrease in total sleep time and sleep efficiency as well as increase in wake after sleep onset and sleep latency (40). Third, gender should also be considered because although slow wave sleep diminishes as one ages, women typically maintain more slow wave sleep than men (40). Fourth, BBF may be efficacious for different sleep disorders. For example, in the case of insomnia, using alpha and theta to induce sleep and a well-designed BBF algorithm tailored to the listener may be beneficial. As for hypersomnia using the theta-alpha-beta structured waking may mitigate this. For those who have circadian rhythm disorders, due to changing work shifts, change in number of hours available to sleep, or traveling between time zones, the use of a full night audio track that can be adjusted for the number hours available to sleep may help realign the circadian rhythm. Fifth, investigators should be careful when considering using BBF with persons with traumatic brain injury as there is no evidence to support that BBF would be helpful or harmful. Finally, consideration should also be taken when using BBF in persons who suffer from serious tinnitus as the ringing in the ears may make listening to the tones uncomfortable.

### Limitations

The use of polysomnography is the gold standard for assessing sleep. However, when multiple data collection points are needed on an intervention that has not been well-studied, it is best to start by conducting assessments with low participant burden, low risk, low cost, and user-friendly and relevant data collection tools. Using a model hypnogram to construct a BBF algorithm may not work for everyone given that each person has their own sleep cycle rhythm, and that age and gender can play a role. However, if changes are noted in sleep parameters such as sleep latency, wake upon sleep onset, or number of times awakened this may provide evidence that the brainwaves can in fact be entrained. Finally, constructing an audio file that allows breaks for REM is a difficult task as sleep cycles are individualized. Using the 90–110-min mark to halt BBF to provide a break is the best educated option aside from simply using continuous theta BBF for the entire night.

## Conclusion

There are a variety of evidence-based strategies to mitigate poor sleep quality, however BBF is one novel idea, that deserves more research. The use of specialized audio frequencies to affect brainwaves to change the listener state of consciousness has proven efficacious in a variety of circumstances. However, BBFs marketed for sleep often only use one frequency (e.g., theta or delta) which may not allow the listener to transition through the phases of sleep. Phase 1, of this four-phase study, concluded that theta BBF was able to decrease stress to help induce sleep. Phase 2 will now assess if theta and delta BBFs, with breaks to allow for REM, will be able to help sustain sleep. Phase 3 will next incorporate theta, alpha and beta BBFs, respectively at the end of an all-night audio track to trigger a structured waking process with Phase 4 combining lessons learned from Phases 1, 2 and 3 to create a customized all-night audio track to entrain sleep. The data obtained from these feasibility studies will provide information to help construct an all-night audio program with the appropriate BBF and timing to trigger the correct sleep stage for better sleep efficiency. If this concept is feasible, it could be useful for many sleep disorders.

## Ethics statement

This study involving human participants was reviewed and approved by the United States Army Medical Research and Materiel Command Institutional Review Board (#M-10544) and the Regional Health Command-Atlantic Institutional Review Board (#RHCA18010/899685). The participants provided their written informed consent to participate in the study.

## Author contributions

MAG designed and conceptualized the study protocol.
